# Migrasomes: Biogenesis, physiological roles, and therapeutic potentials

**DOI:** 10.1083/jcb.202403051

**Published:** 2024-10-14

**Authors:** Haifeng Jiao, Li Yu

**Affiliations:** 1 State Key Laboratory of Membrane Biology, Tsinghua University-Peking University Joint Centre for Life Sciences, Beijing Frontier Research Center for Biological Structure, School of Life Sciences, Tsinghua University, Beijing, China

## Abstract

Migrasomes, vesicular structures discovered in migrating cells, arise from the junctions or tips of retraction fibers, and gradually grow to microscale vesicles. Migrasomes have garnered attention for their role in intercellular communication and potential therapeutic implications. This review presents an overview of recent advances in migrasome biology, covering the mechanisms of migrasome biogenesis, essential physiological roles, and their association with various diseases, alongside potential therapeutic applications. Furthermore, we share our perspectives on potential future directions in the study of migrasomes and highlight the challenges that remain in this developing area of research.

## Teaser

Jiao and Yu review the mechanisms of migrasome biogenesis, physiological functions, as well as roles in various diseases and therapeutic potentials.

## Introduction

In the Tanzanian grasslands of East Africa, wildebeests leave tracks on the terrain as they migrate 3,000 km across the Serengeti Plain; ants searching for food in the backyard leave behind scents as they wander around; in our own house, we leave fingerprints and scents unique to us on whatever you touch. It seems that wherever life travels, it tends to leave its traces behind.

This also applies to the microscopic world. In 2015, we reported the discovery of large vesicular structures, ∼2 µm in diameter, produced by migrating cells at the intersections or tips of retraction fibers (RFs), which are elongated membrane tethers extending from the rear of migrating cells. Inside these vesicles are numerous small vesicles with a diameter of about 50 nm. The formation of these structures is migration-dependent, thus, these structures are named migrasomes. Once formed, migrasomes are left behind when cells migrate away; the detached migrasomes are either ruptured, thus releasing their contents into the extracellular space, or engulfed by other cells, thus transferring the material to the engulfing cells (Ma et al., 2015). While the physiological roles of migrasomes were not known at that time, these observations led us to propose that migrasomes may be an organelle for cell–cell communication.

Over the past decades, a variety of methods have been developed to label, observe, isolate, characterize, and analyze migrasomes both in vivo and in vitro. These methods include probes and tagged proteins for labeling migrasomes (Chen et al., 2019; Huang et al., 2019; Liang et al., 2023; Ma et al., 2015), advanced microscopy techniques for long-term in vivo observation with minimal phototoxicity (Lu et al., 2024; Wu et al., 2021), centrifugation-based and affinity-based purification protocols for isolating and enriching migrasomes from biological samples (Jiang et al., 2019; Jiao et al., 2024; Ma et al., 2015; Wang et al., 2022; Zhao et al., 2019), and the identification of marker proteins that distinguish migrasomes from other types of extracellular vesicles (EVs) (Wang et al., 2022; Zhao et al., 2019). The advancement of these techniques has significantly enhanced researchers’ ability to explore migrasome biology in depth.

It is now evident that migrasomes have roles both within and outside the cell. Once formed, migrasomes can remain attached to the cell for several hours. During this phase, they serve as sites for localized exocytosis, where secretory proteins, including cytokines and chemokines, are transported to and released from the migrasome (Jiao et al., 2024). After detachment, migrasomes become EVs and perform their functions remotely. Migrasomes are characterized by distinct morphology, composition, biogenesis pathways, and marker proteins compared with other EVs, such as exosomes (Ardalan et al., 2022; Arya et al., 2024; Ding et al., 2023; Dixson et al., 2023; Han et al., 2022; Harding et al., 2013; Huang et al., 2019; Jiao et al., 2021, 2024; Liang et al., 2023; Ma et al., 2015; van Niel et al., 2018, 2022; Wang and Yu, 2024; Wu et al., 2017; Zhao et al., 2019; Zhu et al., 2021) (Table 1).

**Table 1.** Differences between migrasomes and exosomes

Property	Migrasome	Exosome
Diameter	∼500–3,000 nm	∼30–150 nm
Cargoes	Secretory vesicles Damaged mitochondria mRNA, protein, lipid, etc.	DNA mRNA, miRNA, Long non-coding RNA (LncRNA) Protein, lipid, etc.
Biogenesis	Migration-dependent ECM-dependent At the junctions or tips of RFs	Migration-independent ECM-independent Multivesicular body
Regulators	Sphingomyelin (SM), SM synthase2 (SMS2) Integrins, PI(4,5)P_2_, PIP5K, Rab35 Tetraspanins, cholesterol, Syt1, etc.	ESCRTs, tetraspanins, ceramide, cholesterol, flotillins Rab7, Rab31, TBC1D2B Rab27, Synaptotagmin-7 Exocyst, etc.
Markers	NDST1, PIGK, CPQ, EOGT, etc.	CD9, CD63, TSG101, Syntenin 1, etc.

**Figure 1.**
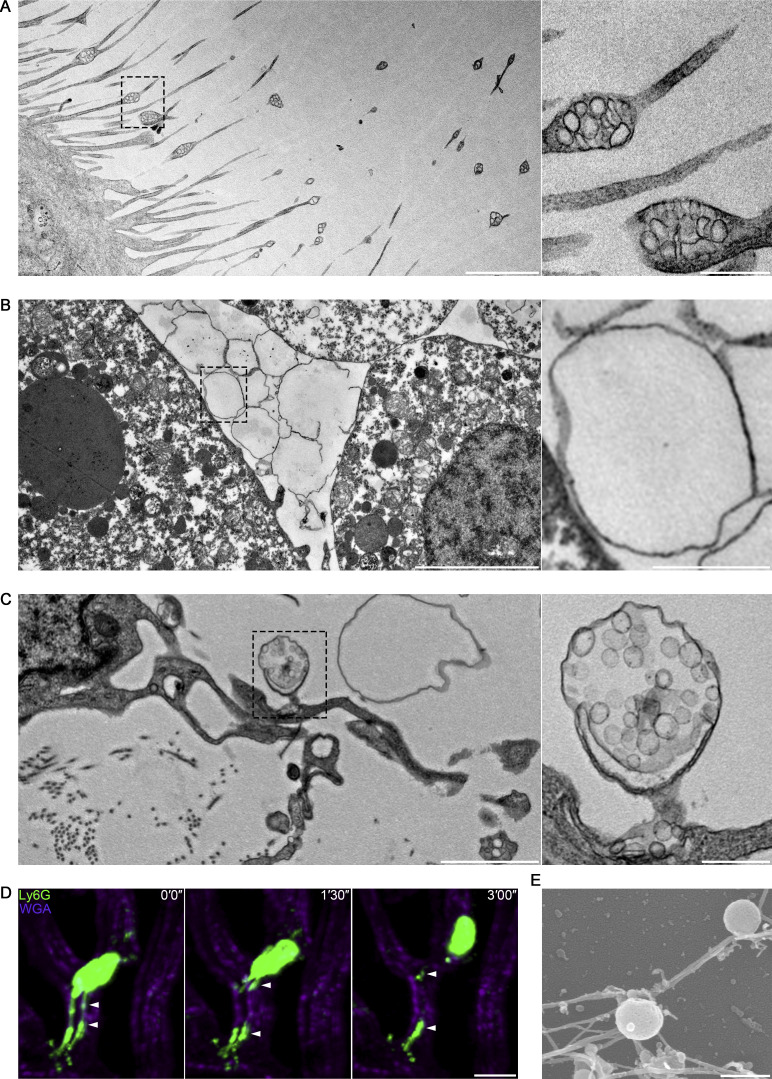
**Migrasomes are widely distributed in many migrating cells in vitro and in vivo. (A)** Representative transmission electron microscopy image of an L929 cell (mouse fibroblast cell line). The right panel shows enlarged pomegranate-like structures, which we named migrasomes. **(B)** Transmission electron microscopy image of a migrasome-enriched pocket between the yolk syncytial layer and mesendodermal cells in a zebrafish gastrula. The right panel shows an enlarged migrasome. **(C)** Transmission electron microscopy image of chicken embryo from day 9 in embryo CAM. The right panel shows an enlarged migrasome. **(D)** Intravital imaging of circulating neutrophil and neutrophil-derived migrasomes in mouse liver. Neutrophil and neutrophil-derived migrasomes are detected with Ly6G-PE antibody. WGA-F647 labels blood vessels and white arrowheads indicate Ly6G-positive migrasomes. **(E)** Representative scanning electron microscopy image of migrasomes isolated from blood neutrophils. Scale bars: 2 μm (left), 500 nm (right) (A); 5 μm (left), 1 μm (right) (B); 2 μm (left), 500 nm (right) (C); 10 μm (D); 500 nm (E).

The biogenesis pathway for migrasomes appears surprisingly complex. Migrasomes are widely distributed in many migrating cells in vitro, such as fibroblast cells (Fig. 1 A), cancer cells, immune cells, and stem cells, among others. Moreover, migrasomes have been observed in various biological settings and have demonstrated important physiological roles in vivo. For instance, migrasomes are formed by mesendodermal cells during gastrulation in zebrafish embryonic development (Jiang et al., 2019) (Fig. 1 B). Similarly, monocytes can release migrasomes during chicken embryonic development (Zhang et al., 2022) (Fig. 1 C). In mouse blood vessels, circulating neutrophils generate migrasomes (Jiao et al., 2021) (Fig. 1, D and E). These studies have elucidated a diverse array of physiological functions for migrasomes, ranging from maintaining mitochondrial homeostasis to establishing specific signaling gradients. Furthermore, there is growing evidence that migrasomes have significant roles in a variety of diseases (Table 2). Consequently, the study of migrasomes is gaining recognition as a nascent field with relevance for researchers across multiple disciplines. This review aims to summarize these developments, offering our thoughts on potential future directions for this burgeoning area of research.

**Table 2.** Distribution and functions of the migrasomes

Cell types or tissues	Contents	Potential physiological and pathological roles	Reference
L929 cells NRK cells	Small vesicles Mitochondria mRNA	Packaged release and localized secretion of signaling proteins Maintaining mitochondrial homeostasis Lateral transfer of materials	Jiao et al. (2021), (2024); Ma et al. (2015); Zhu et al. (2021)
Mouse monocytes	Small vesicles	Packaged release and targeted delivery of cytokines at inflammation sites	Jiao et al. (2024)
Mouse neutrophils	Mitochondria	Maintaining mitochondrial homeostasis	Jiao et al. (2021)
Mouse macrophages	Small vesicles Mitochondria	Packaged release and localized secretion of signaling proteins Maintaining mitochondrial homeostasis	Jiao et al. (2021); Ma et al. (2015)
Zebrafish embryos	Chemokines	Organ morphogenesis during zebrafish gastrulation	Jiang et al. (2019)
Chick embryo AM	Growth factors, chemokines	Angiogenesis in chicken embryos	Zhang et al. (2022)
Tumor cell lines	Cytoplasm, mRNA, etc.	Mediating the tumor–tumor associated osteoclast coupling	Gu et al. (2024)
BM-MSCs	Dermcidin	Enhancing bacterial phagocytosis of pulmonary macrophages	Li et al. (2023)
Multipotent MSCs	SDF-1	Chemoattractant for cells of hematopoietic cells	Deniz et al. (2023)
Cells infected with viruses	Virions	Viral transmission	Liu et al. (2023); Lv and Zhang (2023); Zhao et al. (2024)
Macrophage lineage cells	CD5L	Impairment of complement-dependent blood–brain barrier in CAA	Hu et al. (2023)
Primary human coronary artery endothelial cells	Unknown	Cell–cell signalization	Gagat et al. (2021)
Retinal pigmented epithelium cells	Unknown	Development of proliferative vitreoretinopathy	Wu et al. (2022)
Microglia	Contractile proteins, etc.	Associated with high-salt diet–induced ischemic brain damage	Schmidt-Pogoda et al. (2018)
Urine	miRNA	Biomarker for early podocyte injury	Liu et al. (2020)

L929 (mouse fibroblast cell line), NRK (normal rat kidney cell line).

Our group also discovered retractosomes, small EVs that originate from the breakage of RFs in cells (Wang et al., 2022). These vesicles are characterized by their round shape and continuous membrane, distinguishing them from mere broken membrane fragments. Retractosomes vary in size from 50 to 250 nm and are found to form in a “beads-on-a-string” structure along RFs. While retractosomes have been observed in various in vivo settings, including in circulating neutrophils in mice and embryonic cells during zebrafish gastrulation, their physiological functions are still largely unknown (Wang et al., 2022). As retractosome formation is contingent on cell migration and is tightly linked to migrasome production, we propose the classification of retractosomes as a specific subtype within the migrasome family.

Although both migrasomes and retractosomes originate from or on RFs and depend on migration, significant differences exist between the two structures. Migrasomes are larger in diameter than retractosome, measuring ∼2 µm (Ma et al., 2015) compared with 50–250 nm (Wang et al., 2022). Secondly, the formation process differs markedly in duration and location: migrasomes require hours to develop and form at the branching points or ends of RFs (Ma et al., 2015), while retractosomes form along the fibers within minutes (Wang et al., 2022). Cholesterol is required for migrasome (Huang et al., 2019) but not retractosome formation, with retractosome production being even enhanced in cholesterol-depleted conditions (Wang et al., 2022). Additionally, migrasomes often contain small vesicles or damaged mitochondria (Jiao et al., 2021, 2024; Ma et al., 2015), while retractosomes lack any identifiable internal structures (Wang et al., 2022). Despite sharing some protein components, migrasomes and retractosomes exhibit distinct protein compositions (Wang et al., 2022), emphasizing the unique nature of retractosomes (Table 3). While retractosomes are categorized within the migrasome family, their recent identification means that knowledge about them is still limited. Therefore, this review will primarily concentrate on migrasomes.

**Table 3.** Differences between retractosomes and migrasomes

Property	Retractosome	Migrasome
Diameter	∼50–250 nm	∼500–3,000 nm
Morphology	String of beads–like	Opened pomegranate–like
Biogenesis	Minutes Along the broken-off RFs Cholesterol-independent	Hours At the junctions or tips of RFs Cholesterol-dependent
Cargoes	Lack any identifiable internal structures	Small vesicles Damaged mitochondria mRNA, etc.
Functions	Unknown	Delivery of signaling molecules Maintaining cellular homeostasis Lateral transfer of materials, etc.

## Migrasome biogenesis

Migrasome biogenesis is significantly influenced by various factors that either directly impact it or modulate cellular migration. The dependence of migrasomes on cellular movement means that any element influencing migration can also affect migrasome production. An shRNA screen of 309 proteins localized to migrasomes revealed that the knockdown of 138 of these proteins reduced migrasome formation, while only 33 of the knockdowns did not affect RF formation, suggesting these cells maintained normal migration capabilities (Liang et al., 2023). Thus, the majority of genes affecting migrasome formation identified in this screening impact migration. This underscores the critical role of cellular migration in migrasome generation.

A study focusing on fibroblast cells expressing Tetraspanin 4 (TSPAN4), a key migrasome membrane protein, revealed that cells exhibiting straight, persistent movement had an increased number of migrasomes at their RFs. This phenomenon is linked to the expansion of the cell’s rear during linear movement, facilitating more RF and thus migrasome formation. Further analysis showed that cells moving both faster and in a straight line generated more migrasomes, attributed to longer RFs. The depletion of vimentin, a cytoskeletal protein not present in RFs, resulted in more frequent turning, slower movement, and, consequently, fewer migrasomes (Fan et al., 2022). These results underscore the importance of migration patterns in migrasome biogenesis, highlighting how persistence and speed of movement influence migrasome formation.

### Molecular regulation of migrasome formation

**Figure 2.**
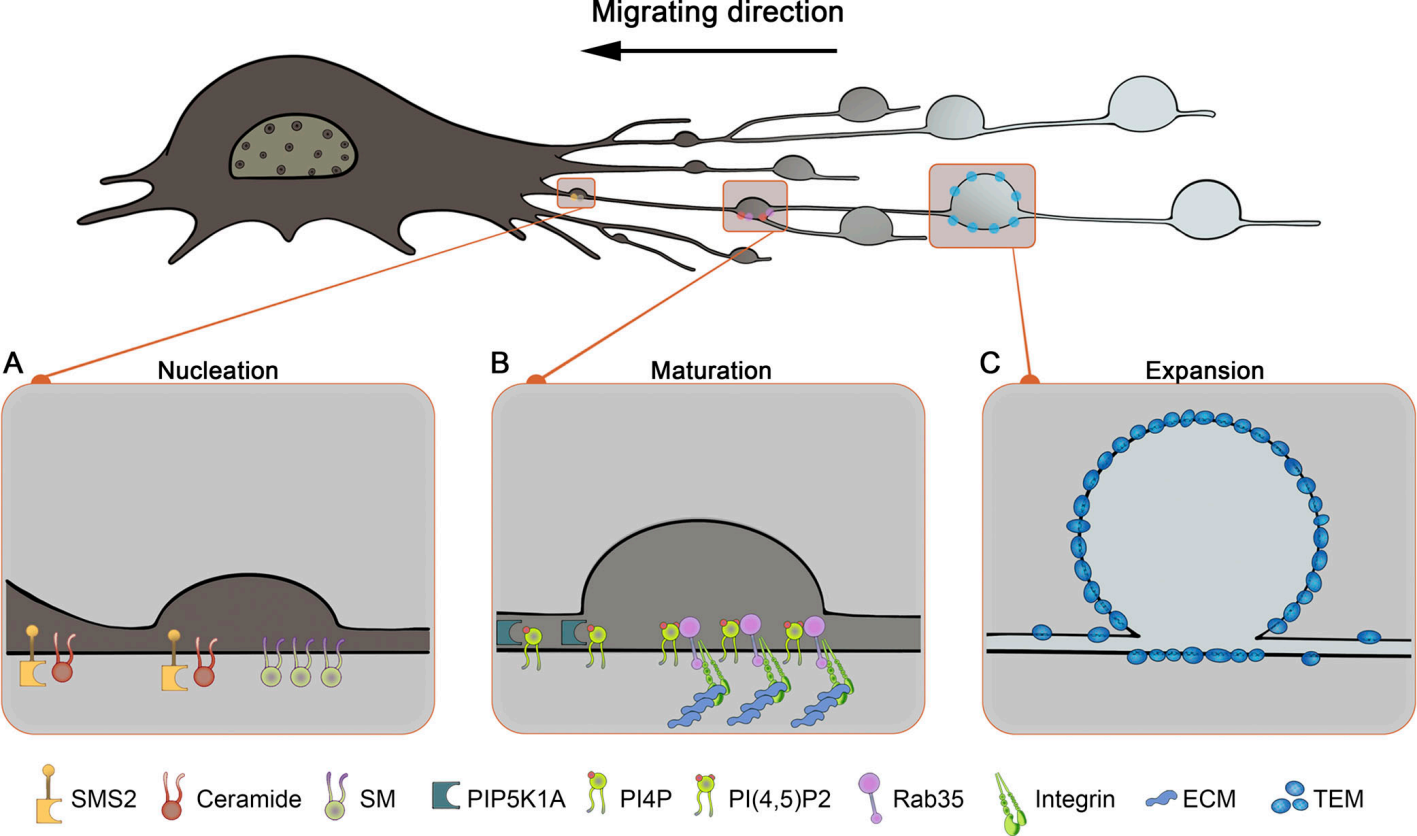
**Categorization of migrasome biogenesis into three phases. (A)** Nucleation; the initial assembly of SMS2 foci marks the first step in migrasome biogenesis. SMS2 will assemble into the SMS2 foci on the basal membrane at the leading edges of migrating cells. These SMS2 foci become more concentrated and remain anchored as the cell migrates, eventually transitioning into RFs and forming MFSs, which determine the migrasome formation sites. **(B)** Maturation; the initial synthesis of PI(4,5)P_2_ and subsequent recruitment of Rab35 and integrin α5 indicate the maturation phase in migrasome development. PI(4,5)P_2_, synthesized de novo at MFSs by PIP5K1A, can recruit Rab35 to MFSs. Through the Rab35–integrin α5 interaction, Rab35 specifically targets and localizes integrin α5 to the bottom of MFSs. This recruitment initiates a tetraspanin-mediated expansion phase. **(C)** Expansion; the recruitment of tetraspanins to MFSs marks the beginning of the migrasome expansion phase. TSPAN4 primarily forms TEMs with cholesterol in the constricted regions on the RF tube. These TEMs are subsequently recruited to swelling regions of RFs, which can be further assembled into larger macrodomains, termed TEMAs. These TEMAs then transition into the vesicle-like structure, typical of migrasomes, to facilitate the final expansion of migrasomes.

Beyond these indirect effects, migrasome formation is directly regulated by a complex molecular machinery. Migrasomes are primarily formed at the branching points or end of RFs. Subsequent investigations have revealed the precursory recruitment of integrins to migrasome sites before TSPAN4, a recognized migrasome marker (Wu et al., 2017; Zhao et al., 2019). This led to the identification of integrin-positive, TSPAN4-negative puncta at RF intersections or ends as migrasome formation sites (MFSs) (Wu et al., 2017). A surprising revelation from a recent study was the extensive biochemical activity at MFSs hours before migrasome biogenesis (Liang et al., 2023), facilitating the categorization of migrasome biogenesis into three phases: nucleation, maturation, and expansion (Fig. 2 and Table 4).

**Table 4.** Overview of key components in three phases of migrasome biogenesis

Migrasome biogenesis	Key components	Reference
Nucleation	SM, SMSs, ceramide	Liang et al. (2023)
Maturation	Integrins PI(4,5)P_2_, PIP5K, PI4P, Rab35	Wu et al. (2017); Ding et al. (2023)
Expansion	Tetraspanins, TEMs Cholesterol Syt1	Huang et al. (2019); Han and Yu (2024)

#### Nucleation

Sphingolipids, in particular sphingomyelin (SM), have a pivotal role in migrasome formation. SM, a major component of plasma membranes, is synthesized from ceramide by SM synthases (SMSs). The two mammalian SMSs, SMS1 and SMS2, are associated with the Golgi apparatus, and both the Golgi and plasma membrane, respectively. Ceramide is produced in the endoplasmic reticulum, transported to the Golgi and then plasma membrane, where it is converted to SM by SMS2 (Huitema et al., 2004).

SM enrichment in migrasomes underscores its vital role in both their formation and structural maintenance. SM depletion using SM hydrolase leads to the collapse of migrasomes, highlighting its structural importance. Importantly, the SM within migrasomes is synthesized de novo at MFSs via SMS2. A particularly fascinating observation is the formation of stationary SMS2 foci on the basal membrane at the leading edges of migrating cells. These SMS2 foci become more concentrated and remain anchored as the cell migrates, eventually transitioning into RFs and forming MFSs. Confocal microscopy reveals that these SMS2 foci on RFs then develop into migrasomes, indicating that at the cell leading edge SMS2 foci formation predetermines migrasome formation (Liang et al., 2023). Thus, the initial assembly of SMS2 foci marks the first step in migrasome biogenesis, serving as a nucleation event that prepares the ground for migrasome formation through subsequent biochemical and biophysical processes (Fig. 2 A).

#### Maturation

Activated integrins appear concentrated at MFSs with the precise pairing of an integrin heterodimer with its specific extracellular matrix (ECM) protein being a key determinant of migrasome formation (Wu et al., 2017). Mechanistically, for RF formation, a portion of the RFs must attach to the cell surface. This attachment serves as an anchor, aiding in pulling the RFs from the rear of the migrating cell. Given the enrichment of integrins on the MFSs and migrasomes, MFSs/migrasomes act as the primary anchoring points for RFs to the ECM. This underscores the dual role of integrins in facilitating cell migration and securing RF attachment to the migrasome.

How are integrins selectively enriched at MFSs? Phosphoinositides, phosphorylated derivatives of inositol phospholipids, have a crucial role in spatially and temporally coordinating organelle biogenesis and functions (Posor et al., 2022). Recent findings underscore the critical involvement of phosphatidylinositol 4,5-bisphosphate (PI(4,5)P_2_) signaling pathways in migrasome biogenesis. PI(4,5)P_2_ is predominantly localized to the plasma membrane, where it is synthesized from PI(4)P by phosphatidylinositol 4-phosphate 5-kinase (PIP5K) enzymes (van den Bout and Divecha, 2009).

PIP5K1A recruitment to MFSs precedes migrasome expansion, and the inhibition of PI(4,5)P_2_ synthesis through PIP5K1A knockdown disrupts migrasome formation. Rab35, a small GTPase that binds PI(4,5)P_2_ (Heo et al., 2006), is recruited to the migrasome by PI(4,5)P_2_. Through the Rab35–integrin α5 interaction, Rab35 specifically targets and localizes integrin α5, which is otherwise uniformly distributed along RFs, to MFSs. This recruitment initiates a tetraspanin-mediated expansion phase. Thus, the initial PI(4,5)P_2_ synthesis and subsequent Rab35 and integrin α5 recruitment delineate a critical maturation phase in migrasome development, facilitating the transition to the expansion phase and underscoring the sophisticated molecular choreography underlying migrasome biogenesis (Ding et al., 2023) (Fig. 2 B).

#### Expansion

After MFS maturation, migrasome biogenesis progresses into the expansion phase. Evidence suggests that this phase is driven by complex biochemical and biophysical processes. Recent studies reveal that migrasome formation is initiated by a biophysical process called tube pearling instability (Dharan et al., 2023; Dharan and Sorkin, 2024). This phenomenon occurs when a cylindrical structure, such as a tube, becomes unstable and forms periodic bulges or “pearls” along its length, typically due to a balance between surface tension and internal pressure or stress. When a tubular membrane experiences a rapid change in tension, it can destabilize the membrane, triggering pearling instability and causing localized swellings. Recent work using living cell imaging and biomimetic systems has shown that a sudden increase in membrane tension can induce localized swelling in RFs, initiating the expansion phase of migrasome formation (Dharan et al., 2023). Subsequently, these unstable swellings are stabilized by multiple mechanisms, leading to further expansion and stabilization of the migrasome.

Recent research highlights the critical role of calcium in migrasome expansion. Synaptotagmin-1 (Syt1), a well-established calcium sensor, is not only enriched in migrasomes but is also essential for their formation in Syt1-expressing cells. The calcium-binding activity of Syt1 is vital for initiating migrasome formation. Recruitment of Syt1 leads to the formation of unstable migrasome precursors, likely through the stabilization of transient bulges caused by tube pearling instability. These precursors are subsequently stabilized via the sequential recruitment of tetraspanins (Han and Yu, 2024).

Tetraspanins, which are transmembrane proteins with four domains, organize into tetraspanin-enriched microdomains (TEMs) within the membrane, each ∼100 nm across (Charrin et al., 2009; Hemler, 2005). TEMs, containing cholesterol and various tetraspanin-associated proteins, are crucial for membrane organization and impact cellular functions such as adhesion, migration, and signal transduction (Hemler, 2003; Zuidscherwoude et al., 2015). TEMs are essential for migrasome formation; cholesterol depletion impairs TEM formation and blocks migrasome production. Similarly, tetraspanins are also critical for the formation of migrasomes (Huang et al., 2019). Out of the 33 identified tetraspanins, the overexpression of 14 significantly boosts migrasome formation, while their knockout impairs it (Huang et al., 2019).

Migrasome formation requires the assembly of these TEMs, found in the RF, into larger macrodomains, termed tetraspanins-enriched macrodomains (TEMAs). These TEMAs then transition migrasomes (Huang et al., 2019). The assembly of TEMAs likely contributes to migrasome expansion by both facilitating expansion and stabilizing migrasomes due to their greater membrane bending stiffness compared with RFs (Dharan et al., 2023; Huang et al., 2019). This analogy resembles a hybrid rubber band composed of both flexible and stiff sections; When stretched, the flexible sections elongate and thin out, while the stiff sections remain unchanged. This causes the stiff sections to protrude relative to the flexible ones, thereby facilitating expansion. In migrasome expansion, these stiff sections correspond to TEMAs. The model proposes that migrasomes exhibit higher membrane bending rigidity than RFs, a theory supported by atomic force microscopy findings (Huang et al., 2019). The increased membrane binding rigidity of TEMs is primarily due to their high cholesterol content, which enhances rigidity by intercalating between phospholipid molecules (Huang et al., 2019) (Fig. 2 C).

The stabilizing role of tetraspanins is demonstrated by recent work using biomimetic systems designed for migrasomes and RFs. The study revealed a two-step process in migrasome development. Initially, swellings, mostly devoid of TSPAN4, form on RFs due to membrane tension within the biomimetic system. These swellings then attract TSPAN4 molecules, leading to expansion into migrasomes. TSPAN4 incorporation is critical for the growth and stabilization of migrasomes (Dharan et al., 2023). A key takeaway is that migrasome expansion is driven not only by biochemical interactions but also by physical factors such as membrane tension, membrane binding rigidity, and mechanical stresses.

## Migrasome functions

Interesting insight into the physiological functions of migrasomes has started to be revealed. Most studies focus on embryonic development, where robust migration and migrasome formation occur. More recently, roles for migrasomes in immune cells such as neutrophils have been described. In this section, we will discuss the physiological roles of migrasomes.

### The fate of migrasomes

Migrasomes, once produced and detached from cells, can follow two distinct destinies. First, they can be internalized by neighboring cells. This process of engulfment by other cells has been documented in the mouse fibroblast cell line L929 (Ma et al., 2015; Zhu et al., 2021). Alternatively, migrasomes may adhere to the surfaces of other cells or the extracellular matrix without being engulfed. Depending on the context, these migrasomes can either remain at their point of origin or be transported to other areas of the organism. For instance, neutrophils produce migrasomes on the surfaces of endothelial cells inside blood vessels (Jiao et al., 2021). These migrasomes persist briefly in this location before the majority, termed neu-migrasomes, are dislodged by blood circulation. During embryonic development, migrasomes created by gastrula cells quickly detach from the cells. These migrasomes then accumulate in various cavities within the embryo, notably beneath the embryonic shield (Jiang et al., 2019). In another context, such as chicken embryonic development, monocytes generate migrasomes in the chorioallantoic membrane (CAM). These CAM-associated migrasomes largely remain stationary at their site of formation (Zhang et al., 2022).

The fate of detached migrasomes influences their functional roles. When engulfed by surrounding cells, the contents of the migrasome are transferred to the recipient cells (Zhu et al., 2021). This indicates that migrasomes can act as vehicles for information and material exchange between adjacent cells. In contrast, migrasomes that are not internalized by other cells can release their contents, affecting neighboring cells (Jiang et al., 2019; Zhang et al., 2022). Notably, when migrasomes stay at their site of origin, they may act as cellular footprints, marking the migration paths of cells. This can provide neighboring cells with information into the movement patterns of the originating cell, potentially playing a role in pattern formation (Zhang et al., 2022).

### Migrasome cargoes

Migrasomes exhibit a unique composition, enriched with specific lipids, proteins, and nucleic acids. Although they originate from the plasma membrane, they contain secretory vesicles from different traffic route (Jiao et al., 2024). As previously mentioned, migrasomes are formed through the assembly of TEMs, which are known to be enriched with specific lipids such as sphingolipids and cholesterol (Huang et al., 2019; Liang et al., 2023; Yáñez-Mó et al., 2009). Additionally, our research indicates that PI(4,5)P_2_ is enriched in migrasomes (Ding et al., 2023). While the full biological implications of these components in migrasome biology are yet to be explored, it’s crucial to recognize that several of these lipids, or their derivatives, act as signaling molecules. For example, sphingolipid metabolites like ceramide and sphingosine-1-phosphate play significant roles in signaling pathways associated with apoptosis, proliferation, stress responses, necrosis, inflammation, autophagy, senescence, and differentiation (Fyrst and Saba, 2010).

Nucleic acids, including minimal small RNA species and selectively enriched full-length mRNA, have been observed to concentrate within migrasomes (Zhu et al., 2021). Additionally, under mild mitochondrial stress conditions, damaged mitochondria can be incorporated into migrasomes; thus, mitochondrial DNA (mtDNA) are also present in these structures (Jiao et al., 2021).

Given that migrasomes derive from the plasma membrane and include secretory vesicles, they potentially encompass three categories of proteins: those from the plasma membrane, cytosolic proteins, and proteins within luminal vesicles. It’s not surprising to find proteins highly concentrated in TEMs, such as adhesion molecules like integrins, also enriched in migrasomes (Wu et al., 2017). Besides membrane proteins, a significant discovery is the high concentration of signaling molecules within migrasomes, including chemokines, cytokines, and growth factors (Jiang et al., 2019; Jiao et al., 2024; Zhang et al., 2022). A recent study has shown that secretory proteins, including signaling molecules, are actively transported into migrasomes via motor protein-mediated transport of secretory carriers, in a manner similar to targeted neurotransmitter release observed in neuronal systems (Jiao et al., 2024). The presence of cytosolic proteins and proteins from various organelles within migrasomes has also been noted, though the mechanisms of their incorporation into migrasomes are still to be determined (Ma et al., 2015; Zhao et al., 2019).

Migrasomes encapsulate various cellular structures, with numerous small vesicles originating from the secretory pathway, being a characteristic morphological feature (Jiao et al., 2024; Ma et al., 2015). Additionally, migrasomes can contain damaged mitochondria (Jiao et al., 2021). Recent studies, particularly in multipotent mesenchymal stromal cells (MSCs), have identified migrasomes that include the late endosomal GTPase Rab7 and the exosomal marker CD63 (Deniz et al., 2023). This observation suggests the intriguing possibility that multivesicular bodies are present within migrasomes.

### Modes of migrasome function

**Figure 3.**
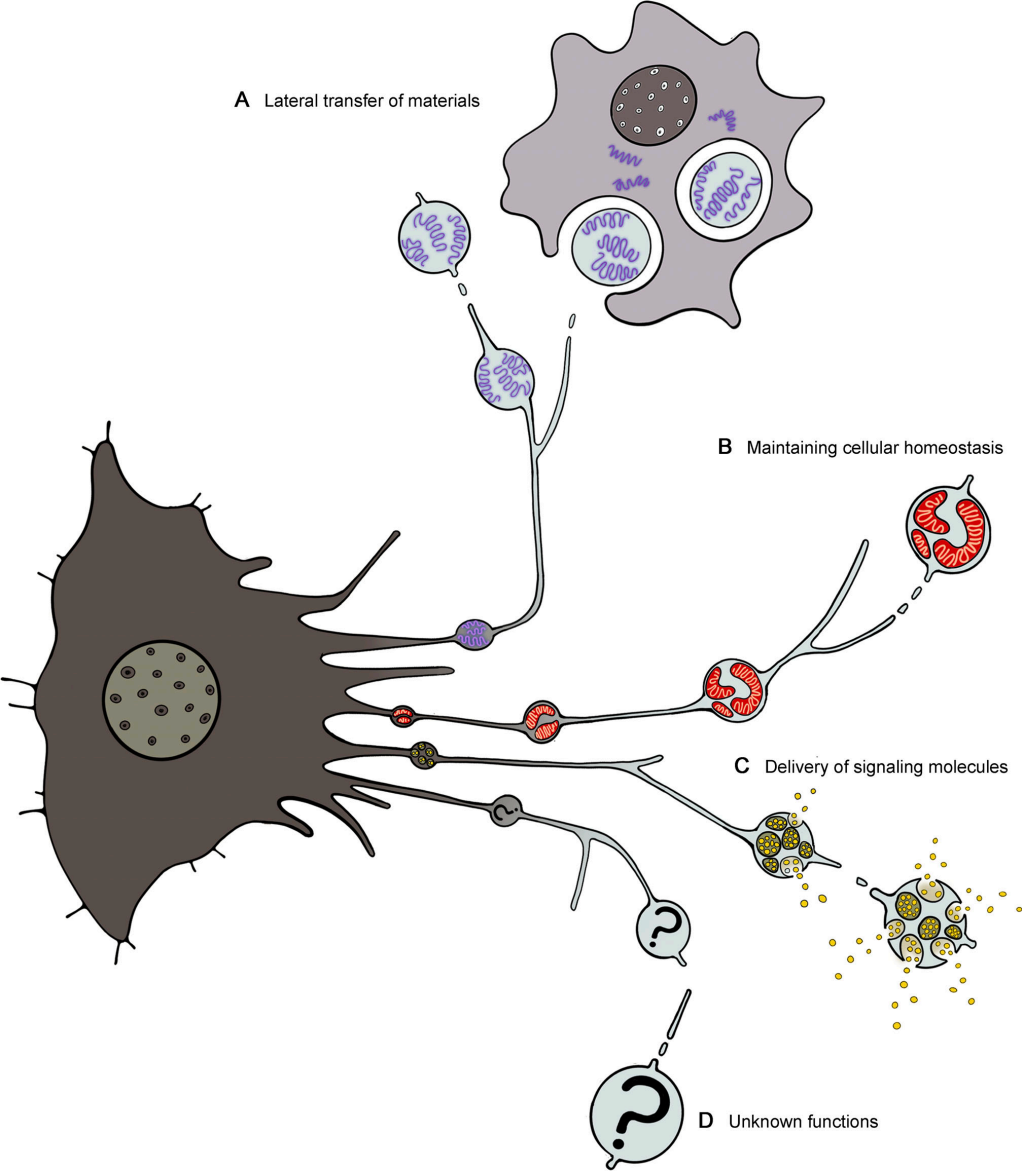
**Modes of migrasome functions. (A)** Migrasomes act as vehicles for material and information exchange between adjacent cells. When engulfed by surrounding cells, the contents of the migrasome such as mRNA are transferred to the recipient cells, affecting neighboring cells. **(B)** Migrasomes mediate the maintenance of cellular homeostasis. Damaged mitochondria can be selectively transported into migrasomes and expelled from cells, in a process termed mitocytosis. Mitocytosis is an important mitochondrial quality-control process in migrating cells, which couples mitochondrial homeostasis with cell migration. **(C)** Migrasomes serve as efficient vehicles for targeted delivery of signaling molecules at specific sites. Signaling molecules such as cytokines, chemokines, morphogens, and growth factors are packaged into migrasomes from various routes. The release of signaling molecules from migrasomes can create migrasome-centered local gradients of these molecules, providing a mechanism to generate localized microgradients. The controlled release of these signaling molecules and spatially defined locations of migrasomes indicate that migrasomes can integrate spatial and biological information. **(D)** Unknown functions of migrasome still await discovery.

Depending on the fate of the migrasome and the nature of its cargos, migrasomes can play various cellular and physiological roles. To date, three modes of migrasome function have emerged: the lateral transfer of material and information between neighboring cells, mitochondrial homeostasis, and the targeted delivery of signaling molecules to spatially defined locations (Fig. 3).

### Lateral transfer of mRNA among neighboring cells

Migrasomes produced by one cell can be internalized by another through processes likely involving endocytosis or phagocytosis, though the exact mechanism of internalization remains to be elucidated (Ma et al., 2015; Zhu et al., 2021). These migrasomes are not just carriers of proteins and lipids but also of mRNA, with a subset of mRNAs being selectively concentrated within them. This specificity suggests a mechanism for sorting these mRNAs into migrasomes. When migrasomes rich in Phosphatase and Tensin Homolog deleted on Chromosome 10 (PTEN) mRNA are introduced to PTEN-deficient tumor cells, they effectively restore PTEN expression, leading to a decrease in Protein kinase B (AKT) phosphorylation and a subsequent reduction in cell proliferation. This indicates that migrasome mRNA can escape the endo/lysosomal pathway, reach the recipient cell’s cytosol, and be translated into functional proteins, thereby altering the recipient cell’s function (Zhu et al., 2021). This finding highlights a mechanism for lateral transfer of material, where active biochemical entities, such as enzymes and signaling molecules, can be transferred between neighboring cells. The occurrence and physiological relevance of this mechanism in living organisms remain subjects for future investigation (Fig. 3 A).

### Maintaining mitochondrial homeostasis

The selectivity of migrasome cargoes extends beyond mRNA. It has recently been shown that damaged mitochondria can be selectively transported into migrasomes and expelled from cells undergoing mild mitochondrial stress, in a process termed mitocytosis (Jiao et al., 2021). This process involves the relocation of impaired mitochondria to the cell’s periphery, driven by their avoidance of inward-directed microtubule-based motor proteins and an increased affinity for outward motor proteins. Mitocytosis plays a vital role in maintaining mitochondrial integrity, protecting cells from the harmful effects of mitochondrial stressors by preserving mitochondrial membrane potential (MMP) and respiration. Physiologically, mitocytosis is vital for maintaining MMP and cell viability in highly migratory circulating neutrophils, highlighting its role as a mitochondrial quality-control process that integrates mitochondrial health with cell migration (Jiao et al., 2021). The coupling between mitochondrial homeostasis and cell migration makes sense, considering migrating cells require more ATP for movement, leading to increased mitochondrial respiration and, potentially, higher ROS production. By removing damaged mitochondria through a migration-dependent mechanism, mitocytosis counteracts the augmented mitochondrial damage, thereby restoring mitochondrial homeostasis. This process may also apply to the homeostasis of other cellular components and structures, a possibility that warrants further investigation (Fig. 3 B).

### Targeted delivery of signaling molecules to spatially defined locations

Besides mRNA and damaged mitochondria, signaling molecules such as chemokines, growth factors, and morphogens are also found to be enriched in migrasomes (Jiang et al., 2019; Jiao et al., 2024; Zhang et al., 2022). Recent research has shown that secretory proteins, including signaling molecules, are packaged into migrasomes via secretory vesicles from both constitutive and regulated secretion pathways. During cell migration, these vesicles become polarized toward the rear of the cell and are actively transported into migrasomes by the actin-dependent motor protein Myosin-5a. Once in the migrasomes, these vesicles undergo local exocytosis through SNARE-mediated mechanisms, similar to synaptic vesicle release. Consequently, migrasomes serve as the primary sites of secretion in migrating cells. Additionally, after detachment, migrasomes packed with signaling molecules act as extracellular carriers, potentially facilitating cell–cell communication. This study reveals the core mechanism driving the accumulation of signaling molecules in migrasomes, detailing how these molecules are enriched within migrasomes and subsequently released (Jiao et al., 2024).

Once formed, migrasomes have been shown to accumulate at spatially defined locations or be deposited along migration tracks in vivo, thus becoming localized sources of signaling molecules. The release of signaling molecules from migrasomes can create migrasome-centered local gradients of these molecules, providing a mechanism to generate localized microgradients (Jiang et al., 2019; Zhang et al., 2022).

Two recent studies focusing on embryonic development emphasize the crucial role of migrasomes in establishing gradients. During zebrafish gastrulation, mesodermal and endodermal cells produce migrasomes that accumulate in embryonic cavities, especially beneath the embryonic shield. These migrasomes, rich in signaling molecules such as chemokines, morphogens, and growth factors, are notably abundant in CXCL12, a key regulator of zebrafish laterality. Kupffer’s Vesicle (KV), a vital organ in zebrafish embryonic development, is instrumental in establishing the body’s left–right asymmetry, essential for the correct positioning of internal organs. This ciliated, fluid-filled structure orchestrates the asymmetric distribution of signaling molecules, guiding the precise orientation of organ development. KV originates from dorsal forerunner cells (DFCs) that migrate and cluster beneath the embryonic shield, guided by their chemoattractant CXCL12. The accumulation of CXCL12-enriched migrasomes in the embryonic shield cavity suggest that these migrasomes act as chemoattractants for DFC migration. Indeed, in *Tspan4*^*−/−*^ zebrafish, where migrasome formation is significantly reduced, DFC clustering beneath the embryonic shield is disrupted, leading to abnormal KV formation and impaired left–right asymmetry. Migrasomes thus regulate left–right asymmetry by establishing a localized CXCL12 source at the embryonic shield cavity, providing localized developmental cues (Jiang et al., 2019). This discovery not only underscores migrasomes’ ability to mediate spatial and chemical information for embryonic patterning but also suggests their wider role in biological processes through the targeted delivery of signaling molecules to the spatially defined location. It should be noted that the chemoattractant function of migrasomes may extend beyond embryonic development. A recent study has found that CXCL12, also known as stromal cell–derived factor 1 (SDF-1), present in migrasomes produced by MSCs, acts as a chemoattractant for co-cultured KG-1a leukemic cells or primary CD34^+^ hematopoietic progenitors (Deniz et al., 2023). This suggests migrasomes could play significant roles in regulating the immune system.

In zebrafish, mesodermal and endodermal cells produce migrasomes that, once formed, detach from the cells and are propelled into the embryonic shield cavity by the embryonic flow. During chicken embryonic development, a different mechanism for migrasome utilization is observed. The CAM, a vascularized structure facilitating gas exchange and nutrient uptake, has been extensively used to study angiogenesis (Nowak-Sliwinska et al., 2014). Recent research demonstrates that highly migratory monocytes traverse capillary formation areas in the CAM, depositing migrasomes along their paths. Contrary to the detachment and dispersal seen in zebrafish, in chickens, these monocyte-derived migrasomes adhere to the ECM, forming migrasome-rich tracks. These migrasomes are packed with key pro-angiogenic factors such as VEGFA and CXCL12, establishing a favorable microenvironment for angiogenesis along the monocytes’ migration routes (Zhang et al., 2022). While this study does not delve into details, it suggests a mechanism for generating intricate spatial patterns, exemplified by capillary networks, through the strategic deposition of migrasomes.

In summary, migrasomes serve as an efficient vehicle for creating localized gradients by concentrating numerous signaling molecules at specific sites. They encapsulate diverse factors in a single structure, enabling complex signal combinations vital for embryonic development. Moreover, the controlled release of these factors, triggered by migrasome-mediated exocytosis or migrasome leakage, allowing precise control over their spatial and temporal distribution. While the study of migrasomes in gradient formation has primarily focused on embryonic development, it’s plausible that this mechanism extends to other areas requiring precise spatial-temporal coordination, such as immune responses and tissue regeneration (Fig. 3 C).

### Migrasomes in pathological conditions

The physiological significance of migrasomes has naturally led to the investigation of their role in various diseases. The burgeoning body of literature since migrasomes’ discovery underscores their association with specific disease states. Initially, migrasomes were identified in the infarcted brain tissues of stroke patients—a finding not observed in healthy brain tissues. This particular study not only established a pathological link but also connected a high-salt diet to increased migrasome formation in mice, suggesting that excessive salt intake may amplify ischemic brain damage by promoting migrasome formation (Schmidt-Pogoda et al., 2018). This revelation positioned migrasomes as potential therapeutic targets for mitigating the effects of acute ischemic stroke.

In the ensuing years, research has expanded to uncover migrasomes in various disease contexts. For instance, migrasomes derived from retinal pigmented epithelium cells have been implicated in the development of proliferative vitreoretinopathy, a condition that can lead to blindness (Wu et al., 2022). In models of cerebral amyloid angiopathy (CAA), Aβ40-induced macrophage-derived migrasomes were found adhering to blood vessels, with an increased capacity for migrasome production by macrophages and the presence of membrane attack complexes in the bloodstream correlating with disease severity in both human patients and Tg-SwDI/B mice models (Hu et al., 2023). Additionally, bone marrow MSCs (BM-MSCs) mitigate bacterial loads in the lungs of stroke model mice by enhancing the phagocytic capabilities of pulmonary macrophages through migrasome release (Li et al., 2023). Moreover, podocytes can release “injury-related” migrasomes, suggesting urinary podocyte migrasomes as a novel diagnostic marker for early detection of podocyte injury (Liu et al., 2020). The role of migrasomes extends into virology, where cells infected with HSV-2 release migrasomes containing HSV-2 virions, facilitating virus transmission to uninfected cells and leading to productive infections (Liu et al., 2023). Poxviruses have also been observed within migrasomes, suggesting a mechanism for these viruses to evade antiviral treatments (Lv and Zhang, 2023). Furthermore, the genes essential for migrasome formation have been linked to various diseases. For example, tetraspanin CD82 levels are related to prognosis in various cancers, including gastric, colorectal, lung, breast, bladder, prostate, and endometrial cancer (Malik et al., 2009). A recent study indicates that TSPAN4 affects glioblastoma progression (Dong et al., 2024). Additionally, other tetraspanins like CD81 and TSPAN9 have been linked to viral infections (Karlas et al., 2010), highlighting the complexity of migrasomes’ roles in health and disease and underscoring their potential as biomarkers and therapeutic targets in a wide array of pathological conditions (Qin et al., 2022).

The study of migrasomes and their roles in disease is still in its early stages, and much remains to be understood about the mechanisms through which they exert their effects in different pathological contexts. As research progresses, targeting migrasomes or their cargo could emerge as a novel therapeutic strategy for various diseases.

### Future directions

Currently, researching migrasomes remains challenging, primarily due to the lack of advanced methodological development. Investigations into migrasomes have relied heavily on imaging techniques. Although various methods for labeling and imaging migrasomes in vivo have been developed, observation windows are limited to a few model organisms like zebrafish, chicken embryos, and mice. The inability to observe migrasomes in deeper tissues significantly hampers our understanding of their biology. Additionally, while migrasomes can be isolated from cultured cells or bodily fluids such as blood or urine, purifying migrasomes from tissue with sufficient purity is still difficult. Even though proteins specific to migrasomes have been identified, these proteins may also be present on other types of EVs or their presence on migrasomes may be cell type specific. Therefore, there is an urgent need to identify a set of universal markers unique to migrasomes across different cells and not found on other EVs. Advancements in purification techniques and the identification of universal migrasome markers are essential for developing reliable, straightforward, and quantitative migrasome detection methods.

Our understanding of the mechanisms and physiological functions of migrasomes is still rudimentary. Regarding the mechanism, only a handful of genes known to regulate migrasome formation have been identified, highlighting the urgent need for genome-wide screening to systematically identify genes involved in migrasome biogenesis. The regulatory mechanisms of migrasome formation, particularly in response to external stimuli, remain largely unexplored. Deciphering these regulatory mechanisms is crucial not only for a deeper understanding of migrasome biogenesis but also for appreciating migrasomes within a broader biological framework. Lipids are key components of migrasomes, with cholesterol, sphingomyelin, ceramide, and PI(4,5)P_2_ playing critical roles in their formation (Ding et al., 2023; Huang et al., 2019; Liang et al., 2023). These lipids are regulated by intracellular lipid metabolism, suggesting that migrasome formation is likely significantly influenced by this metabolic regulation. However, the impact of intracellular lipid metabolism on migrasome biology has not yet been extensively studied. Future research in this area could not only clarify the mechanisms underlying migrasome biogenesis but also uncover unexpected consequences of lipid metabolism. Additionally, while various specific cargos have been discovered within migrasomes, indicating a selective sorting and transport process, the underlying mechanisms of this selectivity are yet to be elucidated. Research on the physiological roles of migrasomes has been mainly limited to the context of embryonic development. However, considering that migration plays a vital role in processes such as immune response, tissue regeneration, and cancer metastasis, investigating migrasomes in these contexts could not only uncover their roles in these processes but also potentially offer new insights into the regulation of these critical biological activities.

Migrasomes have been identified in various disease contexts, found in tissues, blood, and urine, with their numbers and potentially their compositions linked to different pathological states. This association suggests that migrasomes could serve as valuable diagnostic and prognostic tools across a broad spectrum of diseases. Furthermore, as our understanding of migrasomes’ pathophysiological roles deepens, it becomes apparent that some diseases may directly arise from migrasome dysregulation. Research into migrasome biogenesis has identified a set of essential genes, many of which encode enzymes amenable to inhibition by small chemical compounds. This insight raises the possibility that targeting migrasomes could become a novel therapeutic strategy for treating a variety of diseases.

Currently, the study of migrasomes is in its nascent stages and the healthy growth of this emerging field hinges on a deeper understanding of its mechanisms, physiological roles, and implications in diseases. Furthermore, the continuous development of reagents and methodologies is crucial. Without a solid grasp of the mechanisms underlying migrasome formation and function, establishing reliable genetic models to investigate migrasomes’ physiological roles remains elusive. Similarly, deciphering migrasomes’ roles in diseases is challenging without a thorough understanding of these physiological functions. Moreover, without appreciating migrasomes’ roles in disease and developing migrasome-based diagnostics or therapeutics, the study of migrasomes risks being relegated to a niche of curiosity rather than an important field of research. As we embark on the second decade of migrasome research, we hope that what began as a beautiful observation will flourish into a field of prosperous and impactful scientific inquiry.
